# Mitochondrial–Endoplasmic Reticulum Interplay: A Lifelong On–Off Relationship?

**DOI:** 10.1177/2515256419861227

**Published:** 2019-07-19

**Authors:** Corina T. Madreiter-Sokolowski, Roland M. Malli, Wolfgang F. Graier

**Affiliations:** 1 Gottfried Schatz Research Center, Molecular Biology and Biochemistry, Medical University of Graz, Graz, Austria; 2 Department of Health Sciences and Technology, ETH Zurich, Zurich, Switzerland; 3 BioTechMed Graz, Graz, Austria

**Keywords:** mitochondria-associated membranes, MAMs, IRE1α, Ca^2+^ transfer, aging, mitochondria–ER coupling

## Abstract

This article comments recent publications that highlight an intriguing importance of specific settings in the interaction between the mitochondria and the endoplasmic reticulum to ensure cell-specific functions like the responsiveness to elevated glucose in pancreatic β-cells. Hence, alterations of the mitochondria–endoplasmic reticulum communications under various pathological conditions like aging or cancer often come with enhanced Ca^2+^ transfer that, in turn, yields stimulation of basal mitochondrial activity to meet the increasing adenosine triphosphate demand of the very cell. Such observations identify mitochondria-associated membranes as potential target for new therapeutic strategies against aging or cancer.

Contact sites between mitochondria and endoplasmic reticulum (ER), stabilized by so-called mitochondria-associated ER membranes (MAMs), are forming highly dynamic signaling hubs to enable locally restricted and protected transfer of lipids and Ca^2+^within these cellular subdomains (Csordas, Weaver, and Hajnoczky, 2018). The intraorganellar Ca^2+^ signaling within the MAMs is subject of situation-initiated alterations of its protein content (Carreras-Sureda et al., 2019) and may also consist of self-regulatory features as ER-derived Ca^2+^ controls the mobility of cristae and, thus, might establish a certain structure for ion transfer (Gottschalk et al., 2018). Notably, imbalances in the well-controlled homeostasis within these regions, induced by changes in the transporter toolkit or alterations in the tethering between mitochondria and ER, have been associated with all types of age-related dysfunction, including neurodegeneration (Paillusson et al., 2016), metabolic and cardiovascular diseases (Thoudam et al.,2016; Tubbs et al., 2018), as well as cancer (Kerkhofs et al., 2018).

In our recent publication (Madreiter-Sokolowski et al., 2019), we reported enhanced ER–mitochondrial tethering in aged, serially passaged porcine aortic endothelial cells (PAECs) in comparison to young, freshly isolated PAECs. Confocal microscopy revealed increased ER–mitochondrial interaction in senescent endothelial cells, resulting in enhanced mitochondrial Ca^2+^ uptake in response to ER Ca^2+^ depletion upon stimulation with inositol 1,4,5-trisphosphate generating agonists. Increased mitochondrial Ca^2+^ levels, in turn, triggered mitochondrial metabolism by boosting the activity of Ca^2+^ dependent dehydrogenases of the Krebs cycle. However, the risk for mitochondrial Ca^2+^ overload-induced apoptosis increased under these conditions while at the same time increased expression of ER stress markers was observed. Consistent with these findings, specific vulnerability of cancer cells to Resveratrol has already previously been linked to enhanced mitochondrial Ca^2+^ uptake caused by increased ER–mitochondrial linkage in cancer cells (Madreiter-Sokolowski et al.,2016).

Recently, the dependency of insulin signaling on functional ER–mitochondria interactions could be demonstrated in mice and humans (Tubbs et al., 2018). Consistent with this report, we could show (Klec et al., 2019) that an ER Ca^2+^ leak, triggered by glycogen synthase kinase 3β-driven phosphorylation of presenilin-1, induces a metabolic preactivation of mitochondria by elevation of mitochondrial Ca^2+^ levels in insulin secreting pancreatic β-cells. Besides the physiological importance for insulin secretion of these findings, these data may further point to the crucial role of ER–mitochondrial interplay in age-related diseases (e.g., diabetes mellitus type 2, Alzheimer’s disease). The enhanced ER–mitochondrial interplay might be crucial for aged cells to compensate age-related cellular dysfunction or inefficiency as well as for cancer cells to meet the increased energy demand due to their high proliferation activity. Nevertheless, increased ER–mitochondrial contact might also evolve an Achilles’ heel for all these cells by inducing the risk for mitochondrial Ca^2+^ overload or excessive production of reactive oxygen species.

Altogether, these findings point out that contact stability between mitochondria and ER may be a double-edged sword and mitochondrial Ca^2+^ homeostasis to be a crucial determinant for the cell’s fate (Figure 1). Understanding dynamics and features of mitochondrial–ER contact sites, including occurrence frequency and intensity, structural flexibility, and different mitochondrial Ca^2+^ uptake routes and their fine-tuning mechanisms under physiological and pathological conditions or aging, might unveil potential drug targets. These might help to find novel treatment strategies against still incurable types of cancer or ways to prevent the development of age-related diseases, such as type 2 diabetes mellitus. Considering recent reports about biphasic changes of mitochondrial metabolism during aging (Baker and Peleg, 2017), the timing of potential medical interventions might be crucial and especially challenging to get identified.

## Figures and Tables

**Figure 1:**
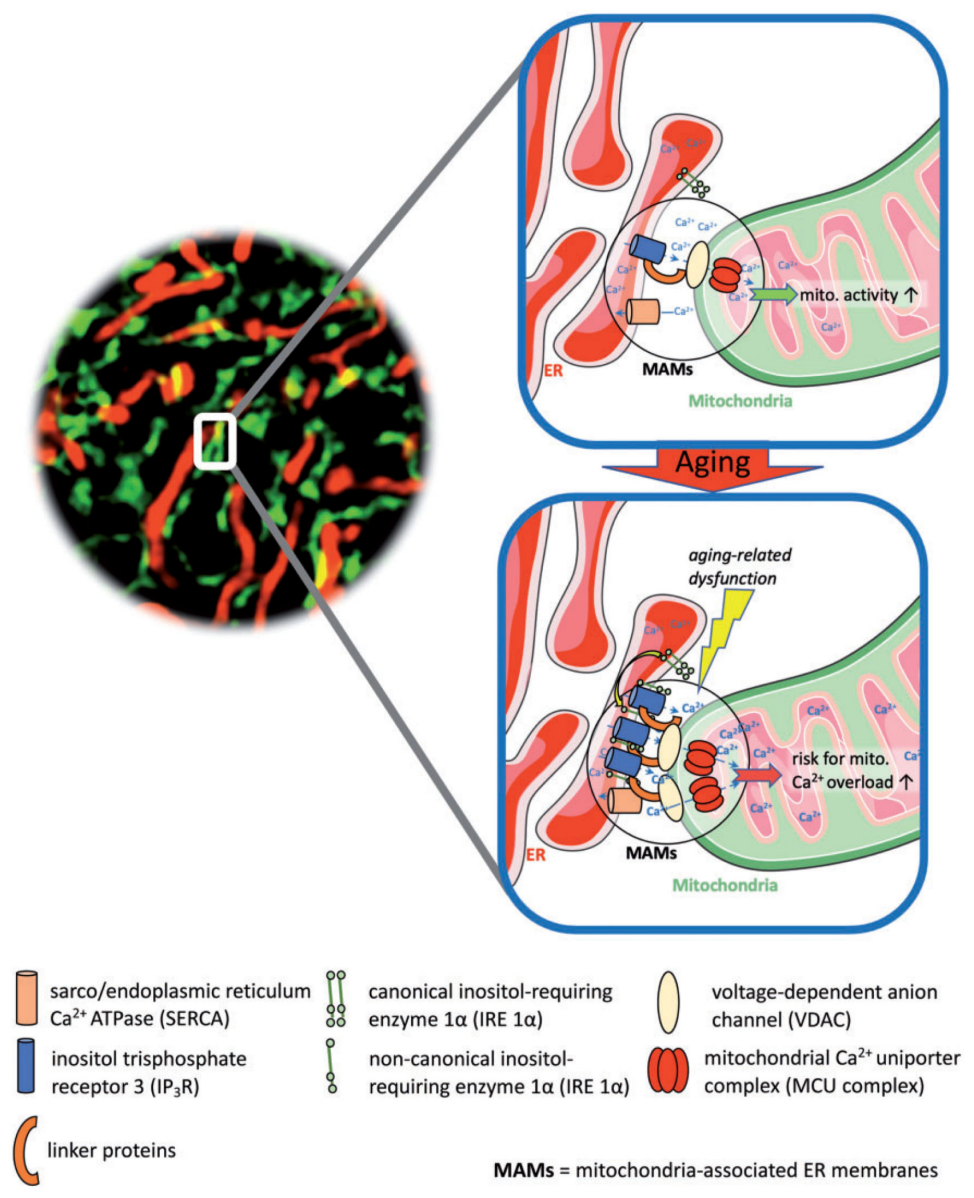
Age-related changes in the assembly and function of proteins in regions of mitochondrial–ER contact sites. Aging-related cellular dysfunction is supposed to cause alterations in the protein assembly, among others trapping of IP3R by IRE 1α within MAM regions, as well as in the ER–mitochondrial tethering, for example, by linker proteins. Thereby facilitated mitochondrial Ca^2+^ uptake via VDAC and the MCU complex might boost mitochondrial activity and help to compensate age-related cellular inefficiency, but also brings the risk for mitochondrial Ca^2+^ overload-induced cell death.

## References

[R1] Baker DJ, Peleg S (2017). Biphasic modeling of mitochondrial metabolism dysregulation during aging. Trends Biochem Sci.

[R2] Carreras-Sureda A, Jaña F, Urra H, Durand S, Mortenson DE, Sagredo A, Bustos G, Hazari Y, Ramos-Fernández E, Sassano ML (2019). Non-canonical function of IRE1α determines mitochondria-associated endoplasmic reticulum composition to control calcium transfer and bio-energetics. Nat Cell Biol.

[R3] Csordas G, Weaver D, Hajnoczky G (2018). Endoplasmic reticulum-mitochondrial contactology: structure and signaling functions. Trends Cell Biol.

[R4] Gottschalk B, Klec C, Waldeck-Weiermair M, Malli R, Graier WF (2018). Intracellular Ca^2+^ release decelerates mitochondrial cristae dynamics within the junctions to the endoplasmic reticulum. Pfluügers Archiv – Eur J Physiol.

[R5] Kerkhofs M, Bittremieux M, Morciano G, Giorgi C, Pinton P, Parys JB, Bultynck G (2018). Emerging molecular mechanisms in chemotherapy: Ca^2+^ signaling at the mitochondria-associated endoplasmic reticulum membranes. Cell Death Dis.

[R6] Klec C, Madreiter-Sokolowski CT, Stryeck S, Sachdev V, Duta-Mare M, Gottschalk B, Depaoli MR, Rost R, Hay J, Waldeck-Weiermair M (2019). Glycogen synthase kinase 3 beta controls presenilin-1-mediated endoplasmic reticulum Ca^2+^ leak directed to mitochondria in pancreatic islets and beta-cells. Cell Physiol Biochem.

[R7] Madreiter-Sokolowski CT, Gottschalk B, Parichatikanond W, Eroglu E, Klec C, Waldeck-Weiermair M, Malli R, Graier WF (2016). Resveratrol specifically kills cancer cells by a devastating increase in the Ca^2+^ coupling between the greatly tethered endoplasmic reticulum and mitochondria. Cell Physiol Biochem.

[R8] Madreiter-Sokolowski CT, Waldeck-Weiermair M, Bourguignon MP, Villeneuve N, Gottschalk B, Klec C, Stryeck S, Radulovic S, Parichatikanond W, Frank S (2019). Enhanced inter-compartmental Ca^2+^ flux modulates mitochondrial metabolism and apoptotic threshold during aging. Redox Biol.

[R9] Paillusson S, Stoica R, Gomez-Suaga P, Lau DH, Mueller S, Miller T, Miller CC (2016). There’s something wrong with my MAM; the ER–mitochondria axis and neurodegenerative diseases. Trends Neurosci.

[R10] Thoudam T, Jeon JH, Ha CM, Lee IK (2016). Role of mitochondria-associated endoplasmic reticulum membrane in inflammation-mediated metabolic diseases. Mediators Inflamm.

[R11] Tubbs E, Chanon S, Robert M, Bendridi N, Bidaux G, Chauvin MA, Ji-Cao J, Durand C, Gauvrit-Ramette D, Vidal H, Lefai E (2018). Disruption of mitochondria-associated endoplasmic reticulum membrane (MAM) integrity contributes to muscle insulin resistance in mice and humans. Diabetes.

